# Role and Mechanisms of Angiogenesis in Tumours

**DOI:** 10.3390/biology14070756

**Published:** 2025-06-25

**Authors:** Aleksandra Sobczyńska-Rak, Beata Żylińska, Beata Nowicka, Eryk Rak, Tomasz Rzepka

**Affiliations:** 1 Department and Clinic of Animal Surgery, Faculty of Veterinary Medicine, University of Life Sciences in Lublin, 20-612 Lublin, Poland; olsob2@gmail.com (A.S.-R.); beatanowicka@aol.com (B.N.); 2 Medical Faculty-Student, Medical University of Lublin, 20-059 Lublin, Poland; e.rak.foto@gmail.com; 3 Veterinary Clinic “PUMA”, 27-400 Ostrowiec Świętokrzyski, Poland; pumawet@interia.eu

**Keywords:** angiogenesis, tumour, vascular sprouting, VEGF, intussusceptive angiogenesis, vasculogenic mimicry

## Abstract

This article presents the accurate and current state of knowledge regarding the process of angiogenesis in neoplastic cells and its importance for tumour development. It provides a detailed review of the different types of angiogenesis with a brief discussion of individual angiogenic factors. Pathological angiogenesis in tumour tissues and the production of angiogenic factors have been recognised as key features responsible for cancerous development and distant metastasis formation since 1971. A phenomenon known as an angiogenic switch allows neoplasm to transform from an avascular to a vascular phase. The emerging new network of blood vessels allows cancer cells to efficiently exchange gases, provide nutrients and eliminate metabolic waste products. The main factors stimulating the angiogenesis process are VEGF, FGF, EGF, PDGF, and TGF-β1. To date, the most specific, strongest and most widely studied factor is VEGF. It is regarded as the main mitogen for vascular endothelial cells, stimulating their proliferation, and it is therefore referred to as a survival factor for cancer cells. Several mechanisms of new blood-vessel formation in cancerous tissue have also been identified. The three dominant processes include vascular sprouting, intussusceptive angiogenesis and vessel co-option. Angiogenesis in cancer tissues remains a subject of numerous scientific studies. A thorough understanding of the mechanism of oncogenesis and tumour expansion appears to be the starting point for future research aimed at finding effective anti-cancer therapy.

## Simple Summary

The treatment of neoplastic diseases is one of the main clinical challenges of modern human and animal medicine. Pathological angiogenesis in tumour tissues and the production of angiogenic factors are recognised as key features responsible for cancer development and metastasis formation. A new network of blood vessels allows cancer cells to efficiently provide nutrients and eliminate metabolic waste products. Thus, the primary tumour can form metastases and spread throughout the body. The main factors stimulating the angiogenesis process are VEGF, FGF, EGF, PDGF, and TGF-β1, with VEGF being the most specific, strongest and most widely studied. The measurement of the expression of pro-angiogenic factors in both humans and animals has diagnostic and prognostic value. Several mechanisms of new blood-vessel formation in cancerous tissue have been identified. The three dominant processes are vascular sprouting, intussusceptive angiogenesis and vessel co-option. Angiogenesis in cancer tissues is still the subject of numerous scientific studies aimed at finding effective anti-angiogenic factors that would inhibit the formation of blood vessels in the tumour as a part of anti-cancer therapy.

## 1. Introduction

The development of neoplasms is a complex process characterised by abnormal signal transmission and cellular metabolism. This leads to the uncontrolled proliferation and inhibition of apoptosis in neoplastic cells. Various types of neoplasms share common features that reflect the universal biological processes involved in the transformation of normal cells into malignant cells. These include mutations in the DNA repair mechanisms, production of growth factors and stimulation of angiogenesis [1,2]. Another key feature is the ability to evade programmed cell death, enabling replicative immortality and resistance to growth-inhibiting signals. The characteristics of the neoplastic process also include the reprogramming of energy metabolism and evading the immune response. The process of pyruvate decarboxylation in mitochondria is a crucial step in cellular energy metabolism. Altered glucose metabolism, however, is a typical feature of cancer cells. These cells, even under normal oxygen conditions, prefer aerobic glycolysis over efficient mitochondrial pyruvate oxidation. This is a phenomenon called the “Warburg effect”. This unusual attribute of tumour cells makes them resistant to oxidative damage and apoptosis, allowing them to proliferate and grow without restriction. Increased glycolytic metabolism also activates proangiogenic pathways, resulting in the increased proliferation of blood vessels within tumour tissues [3].

Neoplastic diseases represent a serious clinical problem in both humans and animals. This fact has prompted many researchers to intensively search for factors influencing oncogenesis, as well as mechanisms of neoplasm development. One of the key features indicating the biological processes involved in neoplastic transformation is angiogenesis. The main purpose of the network of blood vessels produced in the process of angiogenesis is to satisfy the metabolic requirements of neoplastic cells [4,5]. As a neoplastic disease progresses, tumour masses grow too large to receive adequate blood supply from existing vessels. This is when the process known as the angiogenic switch occurs, where the increased production of pro-angiogenic factor initiates the formation of new capillaries based on the existing network of blood vessels [6,7,8]. In addition to providing neoplastic cells with essential nutrients and oxygen, the blood vessels formed also significantly contribute to the development of metastases and tumour expansion.

## 2. Overview

This article attempts to review the scientific literature from 2001 to 2024. The aim is to provide information on the different types of blood-vessel formation in tumours in a process known as tumour angiogenesis.

A literature search was undertaken using Scopus, the Web of Science and search terms such as tumour angiogenesis, vascular sprouting, intussusceptive angiogenesis, vessel co-option, vasculogenic mimicry, glomeruloid angiogenesis and endothelial progenitor cell recruitment. The Pubmed database was also used, which includes primarily the MEDLINE database of references and abstracts on life sciences and biomedical topics.

The literature search was repeated several times. Additionally, relevant original articles identified through the reference list of other publications (e.g., reviews) were included. Even though the literature search was performed without any restrictions on the year of publication, the oldest eligible articles identified were published in 1971, 1997 and 1999. However, the literature published after 2000 was also included, with 48 publications from 2001 to 2014 and 61 publications from 2015 to 2024.

## 3. Molecular Pathways

### 3.1. Physiological Angiogenesis

In both human and animal organisms, the cardiovascular system develops in early embryogenesis, in the process of vasculogenesis. This is the process of forming blood vessels “de novo” from angioblasts that give rise to epithelial lineage, i.e., endothelial precursor cells (EPC). These cells differentiate, migrate and proliferate to form the primary vascular plexus (PVP). To enhance the efficiency of PVP, the angiogenesis process, which involves the formation of blood vessels using a pre-existing vessel, is initiated [9].

Angiogenesis is a strictly regulated phenomenon in terms of both location and duration. Under physiological conditions, it plays a significant role during embryo implantation, lactation, placenta formation and the maturation of ovarian follicles and the corpus luteum, as well as during intense physical activity and in improving organ perfusion [1,10]. The mechanisms of angiogenesis are also involved in wound healing and inflammation.

### 3.2. Angiogenesis in Neoplasms

The process of pathological angiogenesis plays a crucial role in neoplasm formation and has been the focus of numerous scientific studies. Researchers primarily investigate the factors that contribute to its activation, as well as the development of targeted anti-angiogenic therapies. Judah Folkman was a leading researcher in neoplastic angiogenesis, and in 1971, he proposed the concept that angiogenesis is essential for the growth of tumours beyond the in situ (pre-invasive) stage. In the initial phase, an un-vascularised tumour has a volume of approximately 1–3 mm^3^ and comprises approximately 10^6^ cells nourished by diffusion from the vessels surrounding the neoplastic structure [4]. Most tumours in the pre-vascular phase can survive for months or even years. The formation of new blood vessels is a key process for several important functions within tumours. This refers to the elimination of metabolic products and the supply of oxygen and nutrients to sustain the tumour cells and facilitate the formation of neoplastic metastases [4,7]. The confirmation of this hypothesis has become the basis for comprehensive research into angiogenesis, pro-angiogenic factors and the determination of their influence on the formation of vessels [6,11].

The invasive growth of a neoplasm is associated with the secretion, by the tumour cells, of angiogenic factors that stimulate endothelial cells to proliferate and spatially organise into new capillaries. The blood vessels improve the oxygen supply to the tumour cells and facilitate the supply of nutrients necessary for their proliferation [12]. In neoplastic angiogenesis, a very important role is played by the so-called angiogenic switch, a phenomenon involving the interaction between the neoplastic cells and the endothelial cells and cellular matrix [6,7,13]. It conditions the transition of the neoplasm from the avascular phase to the vascularised phase [5,8]. This process is possible thanks to the disproportion of the expression of factors inhibiting and stimulating angiogenesis in favour of pro-angiogenic factors [14]. This marks a turning point for neoplastic transformation, resulting in the development of a network of blood vessels that penetrate the tumour. The nourished cells then begin to proliferate intensively. In the neoplasm, the pro-angiogenic factors, such as VEGF (vascular endothelial growth factor), FGF (fibroblast growth factor), PDGF (platelet-derived growth factor), TGFβ (transforming growth factor β) and TNF (tumour necrosis factor), secreted by endothelial cells and neoplastic cells, begin to predominate [15]. In the angiogenic shift, a very important role is also played by metalloproteinases (matrix metalloproteinases, MMPs), which cause the degradation of the extracellular matrix (ECM) surrounding the vessels and the basement membrane, on which the endothelial cells are located [16]. This enables endothelial cell migration and causes the release of pro-angiogenic cytokines associated with ECM [17]. The migration of endothelial cells is also possible thanks to the expression, on their surface, of adhesive molecules (integrins αv β3, αv β5, selectins E), which interact with ECM components [18].

Other angiogenesis-inducing factors include hypoxia and the increased production of nitric oxide by vascular endothelial cells [15]. A common characteristic of the tumour microenvironment is hypoxia. It is also the most important factor that induces the malignant transformation of neoplastic cells [15,19]. Hypoxia causes a series of changes, including metabolic changes, immune escape and the stimulation of angiogenesis in the tumour [20]. Hypoxia of neoplastic cells leads to the activation of transcription factors, such as HIF-1α and NF-κβ. The hypoxia-inducible factor (HIF-1α) acts on both the endothelial cells and the cells that produce it, including neoplastic cells [6,16,21]. It activates the genes encoding proteins of pro-angiogenic factors and their receptors, including PDGF-B, FGF, EGF (epidermal growth factor) and the main initiator and stimulator of angiogenesis, namely VEGF/VPF (vascular endothelial growth factor/vascular permeability factor) [12,21].

In addition, hypoxia has a significant impact on neoplastic progression, as it causes genomic instability and the emergence of new genetic variants of neoplastic cells [22]. This enables neoplastic cells to move independently [23]. Moreover, the phenotypic characteristics specific to the stem cells, i.e., the resistance to pro-apoptotic signals and ageing signals (senescence) and the capacity for self-renewal also become apparent [24].

### 3.3. Factors Regulating the Process of Neoplastic Angiogenesis

Neoangiogenesis in the body is strictly controlled by stimulating and inhibiting factors. The speed of the process in different tissues is precisely modulated by the interaction between pro- and anti-angiogenic molecules. These factors co-exist in the same microenvironment and remain in a dynamic equilibrium (Table 1).

The predominance of pro-angiogenic factors as a result of reactions to hypoxia, hypoglycaemia, mechanical trauma, inflammation or genetic mutations leads to the formation of new blood vessels [6,10,17].

The factors influencing angiogenesis can be of endocrine (from the circulation), paracrine (from neoplastic cells, stroma, extracellular matrix or macrophages) or autocrine (secreted by endothelial cells) origin, yet the primary target of their action is, in each case, endothelial cells. Most pro-angiogenic factors are found in the extravascular space. In contrast, almost all factors inhibiting the formation of blood vessels have an endocrine effect and influence the cells that circulate through the body with the peripheral blood, controlling neovascularisation [10,25,26].

The predominance of angiogenesis activators has been shown to condition the formation of new blood vessels, whereas the predominance of inhibitors causes “angiogenic silence” and even the regression of blood vessels. The lack of angiogenesis activators, just like the lack of contact with the extravascular matrix, introduces endothelial cells to the apoptosis pathway [12].

Angiogenesis-stimulating factors have a rather broad spectrum of action, yet their most important characteristics include

- A specific effect on endothelial cells, meaning that their appearance induces angiogenesis;
- The presence of specific receptors for these factors on endothelial cells;
- Their disappearance inhibits angiogenesis [8].

The strongest, most specific and most thoroughly researched factor that fulfils all of the above conditions is VEGF [8,11,27,28].

#### 3.3.1. Vascular Endothelial Growth Factor (VEGF)

Vascular endothelial growth factor is found in the form of a glycosylated homodimer with a molecular weight of 46–48 kDa [29]. The VEGF group includes VEGF-A, VEGF-B, VEGF-C, VEGF-D and VEGF-E, which are structurally related but which differ in their biological activity and the placenta growth factor (PIGF) [8,14,25,29]. The most active and researched factor is VEGF-A.

In the process of alternative mRNA maturation, several different VEGF-A isoforms are formed, of which the most important for humans are VEGF_121_, VEGF_145_, VEGF_165_ and VEGF_206_ [6,18,30]. In terms of quantity and biological activity, the dominant form of VEGF-A is VEGF_165_, which is overexpressed in many neoplasms in humans and animals and often correlates with the progression and invasiveness of neoplastic cells [28,29,31]. VEGF is synthesised by various cell types: mast cells, macrophages, fibroblasts, smooth muscle cells of blood vessel walls, neoplastic cells, endothelial cells, monocytes, keratinocytes, eosinophils and lymphocytes T [11,29].

Vascular endothelial growth factor is bound by at least three various types of receptors: VEGF-R1 (Flt-1), VEGF-R2 (KDR/Flk-1) and VEGF-R3 (Flt-4), which belong to the family of receptors containing a domain with tyrosine kinase [6,8,14,18].

VEGF-R1 is found in the endothelium as well as on the surface of macrophages and monocytes. The lack of this receptor has been proven to cause disturbances in the structure and morphology of the resulting vessels.

VEGF-R2 is expressed in endothelial cells, platelets and haematopoietic cells. In animals lacking this receptor, there are disturbances in the formation of vascular endothelial cells and haematopoietic precursor cells. This receptor exhibits a lower affinity for the ligand than VEGF-R1.

The Flt-1 and KDR/Flk-1 receptors differ in their signal transduction mechanisms. The stimulation of the KDR/Flk-1 receptor triggers a strong reaction, whereas the stimulation of the Flt-1 receptor produces a weaker response. This suggests that the Flt-1 receptor can negatively regulate the process of angiogenesis [14,32,33]. In contrast, the activation of the KDR/Flk-1 receptor increases the proliferative activity of endothelial cells while, at the same time, inhibiting the apoptosis process and increasing the permeability of blood vessels. This receptor is probably responsible for the full spectrum of VEGF angiogenic activity [33,34].

VEGF-R3 is detectable in embryonic endothelial cells. In mature tissues, it is primarily found in lymphatic vascular endothelium, which indicates its involvement in lymphangiogenesis [14,32,34].

Even though VEGF receptors are endothelial cell receptors, they have also been found in neoplastic cells, and the VEGF originating from neoplastic cells acts as an autocrine factor that regulates the proliferation, migration and invasion of malignant cells, as well as their survival [15,33]. The most important factor influencing VEGF expression is hypoxia. Under conditions of reduced oxygen partial pressure, the amount in the cell of hypoxia-induced transcription factor HIF-1α (hypoxia-inducible factor), which activates the VEGF gene promoter, increases rapidly [15,18]. VEGF expression can also be stimulated through the influence of other cytokines: EGF, TGF-β, PGF_2_, IGF-1, IL-1 and IL-6 or due to mutations of certain genes (p53, p73, VHL (von Hippel Lindau) or oncogenes leading to neoplastic transformation [15].

Numerous studies confirm that VEGF is the most important mitogen for vascular endothelial cells and that it stimulates their proliferation and migration [6,11,15,28,35]. It is, therefore, a factor responsible for the formation of new vascular structures in neoplastic tumours [11,36]. VEGF is also referred to as a survival factor for endothelial cells and neoplastic cells themselves. It provides newly formed vessels with protection against apoptosis through the direct production of the anti-apoptotic protein Bcl-2 [37]. VEGF increases the permeability of blood vessels being formed. By activating proteolytic enzymes, it actively participates in the destruction of the extracellular space. This creates a space in which new vessels can form [7,15,38].

Very important growth factors influencing the process of neoangiogenesis are also as follows.

- Fibroblast growth factor (FGF);
- Epidermal growth factor (EGF);
- Platelet-derived growth factor (PDGF);
- Transforming growth factor (TGF-β1).

#### 3.3.2. Fibroblast Growth Factor (FGF)

Several FGF forms are distinguished: a-FGF, b-FGF, FGF-3, FGF-4, FGF-5, FGF-6, FGF-7, FGF-8, FGF-9 and FGF-10. In the formation of vessels, the greatest importance is attributed to b-FGF [6,39].

It is secreted by endothelial cells and plays a regulatory role in the recruitment of smooth muscle cells, as well as the migration and sprouting of endothelial cells during the formation of the vessel’s lumen [14]. FGF exhibits synergistic activity with VEGF in the process of vessel formation, both under in vivo and in vitro conditions [39]. FGF stimulates the synthesis of urokinase and metalloproteinases that degrade the basic component of the blood vessel basement membrane, namely type IV collagen. The destruction of the basement membrane facilitates the migration of endothelial cells. The FGF factor also stimulates the proliferation and migration of fibroblasts and induces the synthesis of fibronectin, collagen, proteoglycans and hyaluronic acid, which play an important role in the formation of new blood vessels [40].

#### 3.3.3. Epidermal Growth Factor (EGF)

EGF is found in the environment of the extracellular matrix surrounding the sprouting vessel, contributing to an optimal microenvironment for migrating cells [41]. It, therefore, plays a very important role in cell growth, differentiation, migration and survival [27,38].

#### 3.3.4. Platelet-Derived Growth Factor (PDGF)

PDGF stimulates the migration of pericytes and their adhesion to the newly formed blood vessels, which enables vascular stability and the survival of endothelial cells [6,8,14]. It also plays a major role in communication between neoplastic cells and other tumour microenvironment cells [42].

Research has shown that by interacting with macrophages, PDGF significantly contributes to the evasion of the body’s immune response by neoplastic cells [43]. PDGF/PDGFR also induces the stimulation of pro-angiogenic factors, such as VEGF and FGF, the proliferation of endothelial cells, and the recruitment of endothelial precursor cells into the vessels [6].

#### 3.3.5. Transforming Growth Factor (TGF-β1)

TGF-β1 is a pleiotropic polypeptide that plays a dual role in carcinogenesis. At the early stages of neoplastic transformation, it acts as a growth-inhibiting factor, and it is then involved in the subsequent growth of the tumour and the acquisition of invasive capacity [27]. For the development of a neoplasm, the effect of TGF β1 on the tumour stroma is also important, as it gains the ability to stimulate the proliferation of both neoplastic cells and the stroma [31]. It is a key factor in enforcing immunological tolerance, and the overproduction of this cytokine protects a neoplasm against the immune system. Furthermore, TGF β1, formed by neoplastic cells, can recruit other stromal cells, such as myofibroblasts and osteoclasts, which promotes tumour spread [44].

It has been demonstrated that EGF and its receptors induce, through the interaction with TGF, the synthesis of VEGF in malignant tumour cells and influence the neoangiogenic process [39].

## 4. Types of Angiogenesis in Neoplasms

**Figure 1:**
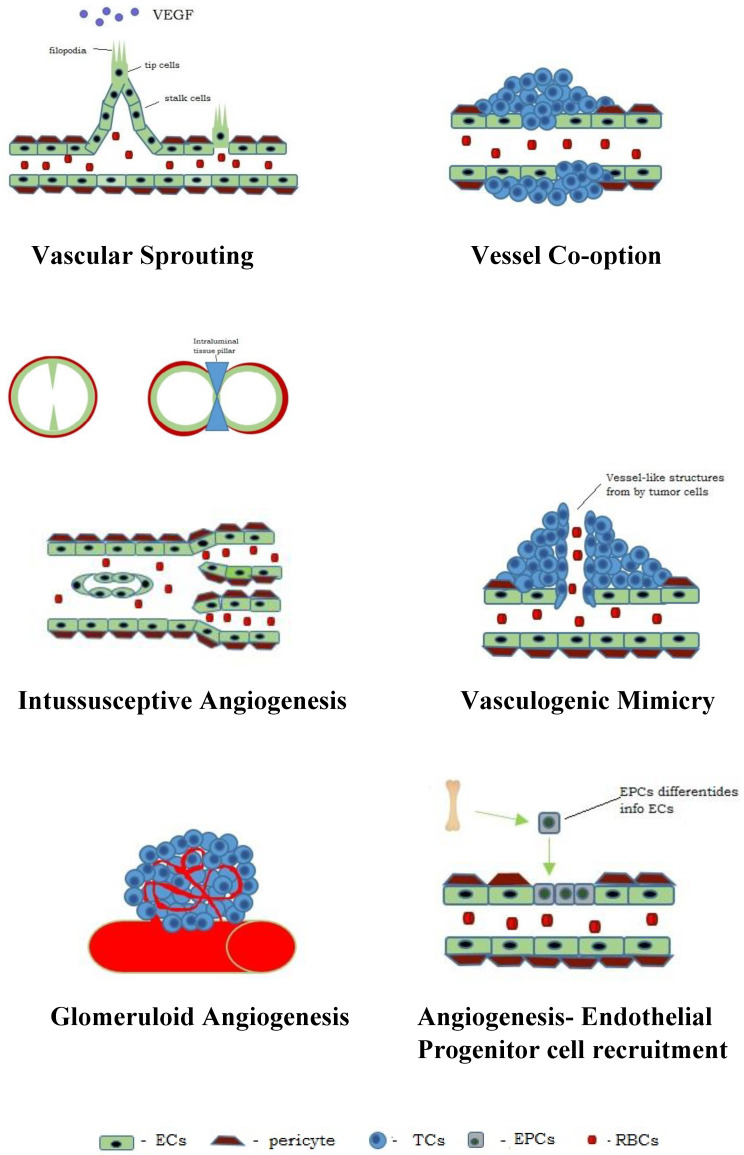
Models of blood vessel formation in tumours—diagram.

During neoangiogenesis, the dominant method of blood-vessel formation is the process of sprouting, intussusception and vessel co-option. It has also been observed that glomeruloid angiogenesis, vascular mimicry and angiogenesis involve the recruitment of endothelial progenitor cells [7,13,39,45,46,47,48,49] (Figure 1).

### 4.1. Vascular Sprouting

The process of vascular sprouting results from the interaction between multiple factors and tumour microenvironment cells. It occurs with the involvement of metalloproteinases (MMPs) and is initiated by the activation of endothelial cells by cytokines and growth factors (VEGF and FGF) produced and released from neoplastic cells [1,49,50]. Under their influence, the structure of the extracellular matrix loosens, facilitating the migration and proliferation of endothelial cells [7,13,20,39].

On the endothelial cells, there are specific receptors for growth factors, which, upon receiving a signal, initiate local remodelling of the extracellular matrix (ECM). There is an increase in the activity of metalloproteinases (collagenases, gelatinases and stromelysins) found in the form of inactive proenzymes in the extracellular matrix and the basement membrane. The process is initiated by cytokines and growth factors that stimulate the secretion of urokinase or the tissue plasminogen activator [51]. The protease plasmin, which is formed from plasminogen, exposes the active sites of metalloproteinases, which initiates the degradation of the main components of the basement membrane and the extracellular matrix, i.e., collagen, laminin and proteoglycans [52]. Consequently, the vascular wall becomes exposed, and the extracellular matrix structure is loosened, creating space for new blood vessels [11,52].

The activated endothelial cells migrate into the newly formed stromal space towards the source of the mitogenic signals [52,53]. Structural and functional heterogeneity has been observed among the migrating endothelial cells. Gerhart was the first to propose the concept of distinguishing leading cells, the so-called “tip cells” and moving cells, the so-called “stalk cells” [54]. The “tip cells” do not proliferate but exhibit the ability to migrate, and their role is to determine the direction of growth of newly formed blood vessels [18,53]. The “stalk cells” are characterised by a high proliferation potential and are involved in the formation of the walls of new blood vessels [55,56,57]. They have VEGF-2 receptors on their surface, which determine their greater dependence on the concentration of VEGF [47]. They are also involved in the formation of intercellular connections and the synthesis of the newly formed basement membrane of the vessel [53].

In the next stage of sprouting, under the influence of VEGF, projections known as “filopodia” emerge on the endothelial cell, which aim towards the VEGF source [54]. The filopodia contains the MTI-MMP (membrane-type metalloproteinase-I) enzyme, which degrades proteins of the extracellular matrix, thus creating space for the growing vessel [8].

The stimulated endothelial cells migrate to the surrounding stroma towards angiogenesis stimulators [11,49]. The “stalk cells” follow the leading cells and branch out and reconstruct the spatial structure of the future vessel with their ability to adhere to each other and the proteins of the extracellular matrix. The endothelial cells elongate and align, thus forming a vascular bundle in which further divisions occur proximally to the migrating tip [30].

In the process of the formation of the lumen of new blood vessels, a significant role, in addition to growth factors (cytokines and interleukins), is played by the following genes: Notch and Coup TFII, as well as EGF [58]. The Notch genes are found in many animal species and play a fundamental role in tissue development and homeostasis. The activation of the Notch pathway is essential to maintain the self-renewal capacity of the stem cells [59]. During the formation of vessels, the Notch signalling system activates the processes associated with cell survival and contributes to the reconstruction, or the so-called vascular remodelling, and the stabilisation of the vascular wall [12,58]. Notch and its ligand DII4, along with the VEGF, control the relationship between the “tip cells” and the “stalk cells” of the endothelium. The Notch receptors also contribute to inhibiting the division of the “tip cells”, whereas VEGF stimulates the division of the “stalk cells” [56,60]. The sprouts grow and, eventually, connect with the existing vessels or other newly formed sprouts [8]. The activated Notch receptors in endothelial cells also gradually suppress cell proliferation [60].

Thanks to their interactions and their capacity to adhere to proteins in the extracellular matrix, endothelial cells recreate the spatial structure of the new blood vessel [11]. The final stages of angiogenesis include the maturation and stabilisation of the vessel [10,13,20,52,59]. Metalloproteinase inhibitors cause the inhibition of the proteolytic phenotype of endothelial cells [8]. Under the influence of PDGF, which binds to the corresponding receptor, periendothelial cells (i.e., pericytes and smooth muscle cells) are recruited. These cells fulfil supporting and stabilising functions in the newly formed blood vessels [50,59].

In the final stage, under the influence of fibronectins, collagen IV, proteoglycans and laminin, the vascular basement membrane is synthesised.

In addition to growth factors, the initiation of the endothelial-cell sprouting process in neoplasms is also influenced by increasing hypoxia. There is overexpression of HIF-1α [22,25], which triggers the expression of subsequent pro-angiogenic factors. HIF-1α also stimulates the expression of endothelial nitric oxide synthase (eNOS). The enzyme causes the breakdown of arginine, which releases nitric oxide molecules that dilate the lumen of blood vessels, thus increasing the efficiency of the angiogenetic process [25].

### 4.2. Intussusceptive Angiogenesis

Another method of angiogenesis is the formation of new blood vessels through the protrusion of connective tissue (transvascular tissue pillar) into the capillary lumen and the splitting of the pre-existing vessel in the so-called intussusception process. The new vascular wall forms within the lumen of the host vessel to form two daughter vessels [61,62]. Tumours use this strategy to quickly adapt to the changing environment [49,63].

The intussusception process is short-lived (from several minutes to several hours), as it requires no proliferation of endothelial cells, basement membrane degradation or interactions with the tissues adjacent to the vessel [25,49]. The process takes place in several steps. It starts with the enlargement of the size of the mother vessel, which results in the endothelial cells being elongated and thinner. The endothelial cells position symmetrically on the opposite walls of the vessel and then form protrusions into its lumen until they come into contact in the so-called “kissing contacts”. The second step involves the reorganisation of inter-epithelial junctions and the rupture of the double endothelial layer. The interstitial tissue is deposited between two layers of endothelial cells, and specific pillars are formed, which become connected over time [64]. The process is accompanied by the movement of pericytes and microblasts, which form vessel-stabilising collagen fibres [9,20]. In the last step of the process, the connective tissue pillars increase their diameter, and the endothelial cells recede, which results in the formation of two separate vessels [64].

The molecular mechanisms involved in intussusceptive angiogenesis are not fully understood, yet this process has been shown to be possibly induced by growth factors, e.g., VEGF, PDGF and erythropoietin [65].

The sprouting and intussusceptive forms of angiogenesis are very often observed at the same time within the same tumour. It has been hypothesised that inhibiting sprouting angiogenesis could stimulate the process of intussusceptive angiogenesis [62]. The fact that intussusception only involves endothelial cell migration and vascular remodelling but does not cause cell proliferation makes it unlikely that anti-proliferative factors would be able to prevent it. In order to develop effective anti-angiogenesis strategies, the new compounds should also include anti-migration characteristics [61].

Histopathological examination of neoplasms, as well as advances in molecular imaging, has provided new data on the growth of tumours and the blood vessels produced. It has been demonstrated that tumours can actually grow without the need to induce angiogenesis [66]. The growth of these tumours is possible because they adapt to the host’s stroma structure and take over the pre-existing blood vessels as a source of tumour vascularisation [63,67]. This form of tumour growth has been observed for the first time in organs with a dense vascular network, such as the lungs, liver and brain. This took place through two main mechanisms: vascular co-option, where neoplastic cells infiltrate and occupy healthy tissues using the pre-existing vessels, and vasculogenic mimicry, in which the neoplastic cells themselves form channels to enable blood flow [68,69,70].

### 4.3. Vessel Co-Option

Many tumours can grow at the avascular stage, mainly in well-vascularised tissues, such as the brain and lungs [61]. This process is referred to as vessel co-option, angiotropism or perivascular invasion [68,71,72].

The essence of the process is to incorporate a pre-existing blood vessel into a tumour [30]. This takes place when a tumour grows along a pre-existing blood vessel and uses it for nourishment without triggering an angiogenic response [71]. Examples include gliomas, which often grow along the vessel walls without forming a capsulated tumour, lung cancer or metastasis into the lymph nodes or the liver [30].

The blood vessels at the interface between the tumour and surrounding tissue may become infiltrated by replacing epithelial cells with neoplastic cells or by neoplastic cells invading the stroma around the blood vessels [73,74].

The molecular mechanisms underlying vessel co-option are not as well understood compared to those of sprouting angiogenesis. It has been demonstrated that adhesion molecules, i.e., integrins and L1CAM, secreted by neoplastic cells, play a major role in the adhesion of neoplastic cells and their spread along blood vessels [19]. Adhesion to the abluminal surface of blood vessels is a critical step during vessel co-option. For example, β1-integrin is essential for the adhesion of cells to the components of the basement membrane of the capillaries in the brain [75].

An interesting phenomenon of vessel co-option has also been observed in the so-called “liquid tumours”. It was found that acute lymphoblastic leukaemia cells use α6-integrin to migrate through the arachnoid vessels into the central nervous system, bypassing the blood–brain barrier [76]. It has also been demonstrated that the involvement of the adhesion molecule L1CAM is an important mechanism for the colonisation of metastases and the spread of neoplasms along blood vessels. This non-angiogenic mechanism of tumour vascularisation appears to be significant in the early stages of growth and the formation of metastases into the brain or liver [73,77].

It was also shown that vessel co-option is a mechanism of resistance to angiogenesis inhibitors and anti-angiogenic therapies [30,74].

### 4.4. Vasculogenic Mimicry (VM)

The phenomenon of vasculogenic, or vascular, mimicry occurs when aggressively growing neoplastic cells form vessel-like structures. During this process, the vascular network is formed without the involvement of specific endothelial cells [47,49,78,79,80,81].

In 1999, Maniotis, Hendrix et al. observed and described the phenomenon of VM for the first time. When examining highly aggressive uveal melanoma cells, they observed the formation of vascular structures that were formed without the presence of endothelial cells lining these channels. In order to distinguish this process from true angiogenesis, they introduced the term “vasculogenic mimicry” (VM) [82]. Since then, the VM concept has been established as an alternative method of vascularisation, which ensures sufficient blood supply and nourishment to the tumour tissue [80,81,83,84].

VM is divided into two separate types: the tubular type and the patterned matrix type. VM of the tubular type is characterised by tubular channels, which consist of an extracellular matrix and are lined with neoplastic cells instead of endothelial cells. VM of the tubular type involves the carving out of pathways by tumours or the invasion of neoplastic cells into the walls of blood vessels [81]. The second type, known as the patterned matrix type, consists of a basement membrane rich in fibronectin, collagen, laminin and heparan sulphate proteoglycans, surrounded by neoplastic cells instead of endothelial cells [85,86].

The characteristics of the structure of the vessels formed in the mimicry process are as follows:

- A lack of vascular endothelial cells on the inner wall of the blood vessel;
- The channels resembling vessels are lined with neoplastic cells;
- The cells lining the channels react positively to PAS staining but negatively to CD31 staining, whereas the endothelial vascular channels are negative in PAS staining but positive in CD31 staining;
- The presence of erythrocytes in the vascular-like channels [87].

VM serves as part of the functional microcirculation. The neoplastic cells lining the inner surface of the channels are directly exposed to the blood flow and can enter the bloodstream more easily [55]. They can also move into the blood vessels lined with endothelium, thus facilitating tumour invasion and metastasis [80,88]. Vascular mimicry probably plays a causative role in neoplastic progression by stimulating the invasive growth and metastasis of neoplastic cells [48,83,89].

One of the major factors involved in the formation of VM is hypoxia and the activation of the HIF [79,81]. Differentiation and remodelling of the microvessels formed in the mimicry process also involve cancer stem cells (CSC), contributing significantly to the invasive nature of neoplasm growth [90,91].

Some authors have reported that VM dominates in early tumour growth, and the vessels lined with endothelial cells are the main microcirculation pattern observed with increasing tumour size [69].

VM represents a challenge for anti-angiogenic approaches (mainly because many VM-competent neoplastic cells have no receptors for typical pro-angiogenic factors, such as VEGF [78,92,93].

### 4.5. Glomeruloid Angiogenesis

Another form of vessel formation in tumours is glomeruloid angiogenesis, which involves the formation of the so-called glomerular bodies (GBs). It is characterised by the migration of endothelial cells towards the VEGF-producing cell and the formation of a bundle of new vessels surrounded by the basement membrane and the adventitia [94]. Glomerular bodies comprise the mother vessel, to which proliferating endothelial cells adhere at the site of a thinned wall that is partially devoid of the adventitia. These bodies grow both in the direction of the extravascular space and towards the lumen of the vessel. The basement membrane of the formed GBs is locally digested, and the pericytes are only found at the periphery of the structure being formed. The ECs growing into the lumen of the mother vessel divide it into smaller channels. Macrophages appear on the edges of the GB structure, yet their role in the process is not yet fully understood [25]. Subsequently, the GB structure is reorganised due to a decrease in VEGF levels. The adventitial cells divide it into smaller structures. As a result of the paracrine effect, some ECs, macrophages and pericytes undergo apoptosis [94]. Under the influence of the PDGF, secreted by endothelial cells, the basement membrane and the extracellular matrix proteins are reconstructed. The final step of glomeruloid angiogenesis is the connection of the vessels by pericytes, which form a structure of clustered capillaries. The blood vessels formed are often too small in diameter, which impedes the flow of blood [95,96]. Under in vivo experimental conditions, the glomerular bodies are visible within the mother vessel, in the vicinity of the neoplastic cell releasing VEGF, as early as after three days [9].

Some researchers are of the opinion that GBs can form passively within a neoplasm as a result of the accumulation of existing capillaries and the branches adjacent to them, which is followed by their remodelling and the formation of daughter vessels [67].

### 4.6. Endothelial Progenitor Cell Recruitment

The formation of vessels that accelerate tumour growth is also induced by the differentiation and recruitment of endothelial progenitor cells (EPCs) or haemopoietic cells originating from the bone marrow [45]. EPCs are mainly unipotent adult stem cells with the capacity for proliferation, self-renewal, involvement in neovascularisation and endothelial repair [41,97]. They were first identified by Asahara et al. [98]. In tumours, neovasculogenesis is initiated by the interaction between the tumour cells and the EPC cells in the bone marrow under the influence of the VEGF secreted in the tumour microenvironment. In addition to VEGF, the mobilisation of EPC to the tumour bed is also influenced by other hypoxia-inducible chemokines [65].

Angiogenic factors released by neoplastic cells can attach to bone marrow-derived circulating EPS, which have VEGFR-2, Tie-2, CD31 and CD34 receptors on their surface and haematopoietic stem cells (HSC) with a VEGFR-1 [99]. As a result of the differentiation or a fusion, the stem cells, with the co-participation of the VEGFR2 of progenitor cells, acquire the characteristics of endothelial cells [2].

It has been demonstrated that EPCs have the ability to migrate to the site of vessel formation, differentiate into endothelial cells in situ, and become a component of the vascular network [46]. The acquisition of progenitor cells is a process comprising the following steps: (1) chemotaxis to VEGF and PIGF; (2) immobilisation within the tumour; (3) migration to the interstitial space with the involvement of selectin and integrins; (4) incorporation into the vessels being formed [9]. The contribution of EPCs to the formation of blood vessels in neoplasms depends on the type of tumour. They are active in breast cancer, whereas their role in the development of stomach cancer or gliomas is insignificant.

Depending on the progression and malignancy of the tumour, neoplasms often use more than one method to form pathological vessels in order to improve vascularisation.

### 4.7. Characteristics of the Vessels Formed During Neoplastic Angiogenesis

Even though the growth of blood vessels during neoangiogenesis is intense, the blood vessels are not fully balanced in terms of function and structure. Due to the significantly shortened life cycle of endothelial cells in the process of intense neoplastic angiogenesis, from 1000 days to 1 week, the vessels in tumours are immature. Their route is chaotic, they are winding and they often have an irregular diameter of the vessel lumen [14,55]. They are often not divided into the arterial, venous and capillary parts, with blind ends and numerous fistulas formed [6,100]. The vascular basement membrane is irregular, thinned and permeable, with smooth-muscle deficiencies present in the vascular walls. Vascular immaturity and the lack of parietal cell association result in their excessive permeability [30,55]. In addition, the vessels in tumours are subject to constant reorganisations, which changes their course and position. This causes extensive transformations within the tumour and changes in the location of necrotic areas [14].

These abnormal features and the slow blood flow in the vessels result in ineffective nutrition of the tissue they supply. This results in chronic hypoxia of the neoplastic cells and continuous induction of the formation of subsequent vessels [15].

## 5. Angiogenesis Assessment

**Figure 2:**
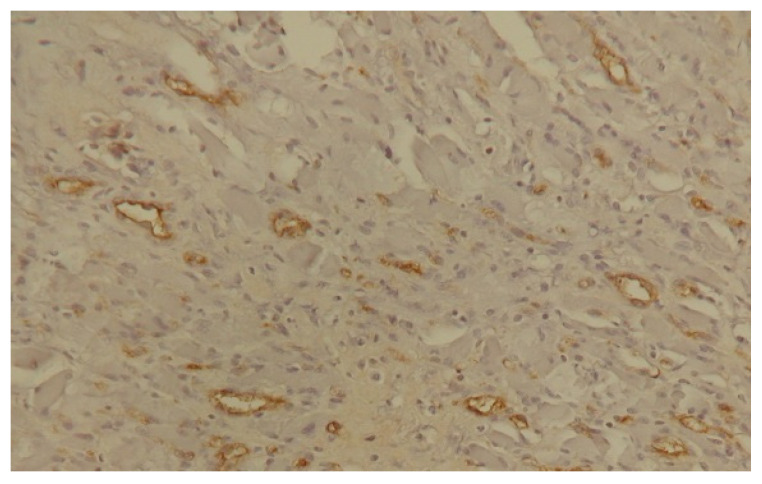
Squamous cell carcinoma—immunohistochemical staining—an anti FVIII antibody (200×).

**Figure 3:**
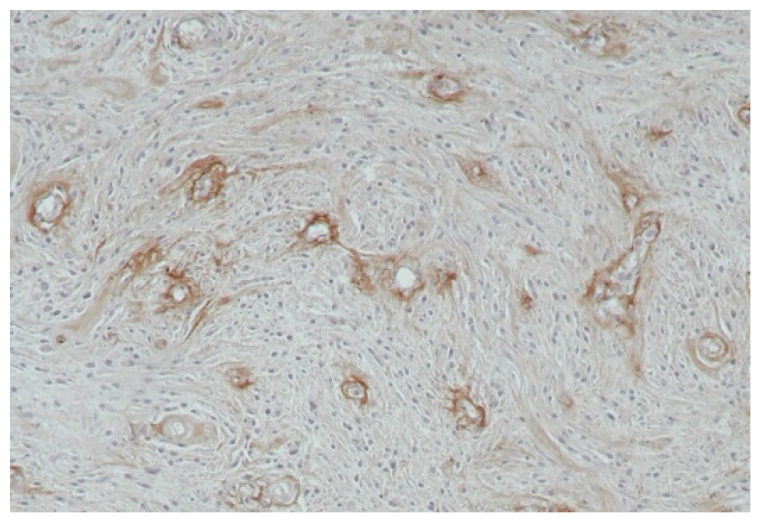
Fibrosarcoma—immunohistochemical staining an anti—FVIII antibody (200×).

In both humans and animals, the vascularisation of tumours is assessed using direct and indirect methods. Direct methods involve the determination of the density of capillaries or endothelial clusters per unit of area of a tissue or organ (MVD—microvessel density). This is enabled by staining the histopathological specimens of surgically removed tumours using immunohistochemical methods [101]. Vascular endothelial cells present several markers that are recognised in immunohistochemical reactions using antibodies. These include the so-called pan-endothelial antibodies, which react with antigens located on the surface of all endothelial cells, and antibodies that bind to antigens found only on the surface of proliferating endothelial cells. Pan-endothelial antibodies include antibodies targeted against von Willebrand factor (VIII) as well as CD31 and CD34 antigens [102] (Figure 2 and Figure 3).

The methods of indirect angiogenesis assessment involve the determination of the production of angiogenic cytokines, their tissue expression, concentration in blood serum and receptor expression. To determine the expression of the factors, Elisa (enzyme-linked immunosorbent assay) immunoenzymatic tests [28,103], as well as spectroscopic methods, are used [104]. In contrast, the expression of the pro-angiogenic factor receptors in the neoplastic tissue is assessed using molecular biology techniques (Western blot and qPCR methods).

## 6. Clinical Relevance

Thanks to numerous studies on angiogenesis and the mechanisms and factors influencing cancerogenesis, it has been demonstrated that it plays a crucial role in the development of cancer, its progression and metastasis. Intussusceptive angiogenesis has been observed in various neoplasms, including melanoma, colorectal cancer, gliomas and breast tumours [62,63,65]. This type of angiogenesis is utilised in neoplasms as a means of rapidly remodelling the vessels and maintaining blood flow within the tumour [65]. Vessel co-option is more frequently observed in tumours of highly vascularised organs, such as the brain, lungs or liver, where the primary neoplastic cells take over the neighbouring, dormant blood vessels found in the tumour tissue.

Vasculogenic mimicry has been observed in numerous neoplasms but mainly in melanomas. It has been demonstrated that the presence of VM in patients with malignant neoplasms is, in many cases, an independent negative prognostic factor. Patients with VM channel-forming melanomas of the uvea and the skin had a worse prognosis and shorter survival [69]. It was also observed in glioma, head and neck cancer, lung cancer, colorectal cancer and prostate cancer, sarcomas and ovarian and breast neoplasms, and it is correlated with high tumour aggressiveness, tumour progression and an unfavourable prognosis for patients [65,70,80,89,92,93].

The VEGF/VEGFR system has been identified as the main angiogenic modulator. Elevated growth-factor levels in both tissues and the blood serum have been noted in many malignant organ tumours in humans and animals [8,31,65,103,105,106,107]. The increased expression levels for mRNA VEGF and its receptors indicated a poor prognosis and the presence of, or tendency towards, metastasis in the neoplasms of the lungs, breast, thyroid, stomach, intestines, oral cavity or the central nervous system [36,107,108]. Elevated levels of this factor have also been noted in haematological hyperplasia. In myeloid leukaemia, this is correlated with a shorter survival period and a lower rate of complete remissions achieved [109].

The indirect determination of angiogenesis by analysing the VEGF levels in the blood serum as well as its receptors can have a prognostic value and is useful in the early diagnosis of malignant neoplasms, including in dogs [28,31,103,105,106,110].

The overexpression of vascular endothelial growth factor, found in malignant neoplasms, in combination with the clinical image of the neoplastic process, can affect the choice of the surgical treatment method and the prognosis of its therapeutic effect. In dogs, VEGFR-2 was detected in SCCs (squamous-cell carcinomas) of the skin [105], malignant mammary gland neoplasms and sarcomas [106,111,112].

The author’s own research into the VEGF and EGF levels in canine neoplasms shows that VEGF stimulates angiogenesis and may serve as a marker in the diagnosis and prognosis of metastatic squamous cell carcinoma of the skin and oral cavity in dogs. In contrast, EGF overexpression occurring in neoplastic processes in the anal region and malignant neoplasms of the oral cavity in dogs correlates with neoplastic progression. The study led to the conclusion that the levels of both VEGF and EGF are good prognostic factors and can be used to monitor oncology patients [28,103].

Currently, many studies are being conducted worldwide that focus on angiogenesis, which undoubtedly promotes the formation of metastases in most types of neoplasms.

The microbiome present in various types of cancers, especially those arising from the mucous membranes, has recently become the subject of research by many oncologists and microbiologists. It has been shown that the tumour microbiome, by activating the local inflammatory response inducing HIF-1 and NF-κβ, leads to the secretion of proangiogenic cytokines, i.e., VEGF, and thus stimulates angiogenesis in cancerous tissues. It has also been confirmed that the tumour-associated microbiota may directly affect oncogenesis, progression and the response to anticancer treatment [113].

Research is also being conducted into the development of medicines containing anti-angiogenic agents, which are intended to effectively inhibit the formation of blood vessels in neoplastic tumours and thus reduce the risk of recurrence and the formation of metastases [14,25].

## 7. Conclusions

Angiogenesis is a process that plays a crucial role in the development of cancer. The newly emerging network of vessels enables not only the nutrition of cancer cells but also the development of metastases. A thorough understanding of the mechanism of vessel formation becomes the basis for new anticancer therapies to intervene at different stages of tumour development. There are several mechanisms of tumour-vessel formation, and this process is controlled by numerous growth factors. The three dominant processes include vascular sprouting, intussusceptive angiogenesis and vessel co-option. Particularly distinctive and interesting forms are glomeruloid angiogenesis and vasculogenic mimicry, with the latter mainly present in melanomas. Depending on the type of cancer and its advancement stage, several methods of vessel formation can occur simultaneously in tumour cells. Therefore, a thorough understanding of the mechanisms of pathological angiogenesis in neoplasms is of primary interest to scientists developing antiangiogenic therapies based on the inhibition of vascular-network formation.

## Figures and Tables

**Table 1:** Angiogenic factors.

Proangiogenic Factors	Antiangiogenic Factors
VEGF—vascular endothelial growth factor FGF—fibroblast growth factor TGF-β—transforming growth factor EGF—epidermal growth factor PDGF—platelet-derived growth factor HGF—hepatocyte growth factor Angiogenin Ang-1—angiopoetin-1 IGF-1—insulin-like growth factor-1 PG-E—prostaglandin-E IL-8—interleukin-8 Proliferin Epo—erythropoetin TNF ά—tumour necrosis factor ά	TIMP—tissue inhibitors of matrix metalloproteinase TSP-1—thrombospondin-1 Angiostatin Endostatin Ang-2—angiopoetin-2 Prolactin (16 kDa fragment) Platelet factors-4 INF α/β—interferon α/β IL-1—interleukin-1 IL-6—interleukin-6 IL-10—interleukin-10 IL-12—interleukin-12 Somatostatin

## Data Availability

Data sharing is not applicable. No new data were created or analysed in this study.
